# The impact of interleukin-6 (IL-6) and mesenchymal stem cell-derived IL-6 on neurological conditions

**DOI:** 10.3389/fimmu.2024.1400533

**Published:** 2024-06-24

**Authors:** Irina Kerkis, Álvaro Prieto da Silva, Rodrigo Pinheiro Araldi

**Affiliations:** ^1^ Genetics Laboratory, Center of Development and Innovation, Butantan Institute, São Paulo, Brazil; ^2^ BioDecision Analytics Ltda., São Paulo, Brazil; ^3^ Post-graduation Program in Structural and Functional Biology, Paulista School of Medicine Escola Paulista de Medicina (EPM), Federal University of São Paulo Universidade Federal de São Paulo (UNIFESP), São Paulo, Brazil

**Keywords:** interleukin-6 (IL-6), mesenchymal stem cells (MSCs), IL-6 dysregulation, neurodegenerative diseases, Huntington disease (HD), stroke

## Abstract

Interleukin-6 (IL-6) is a versatile cytokine crucial for immune response modulation, inflammation regulation, and various physiological processes in the body. Its wide-ranging functions underscore its importance in maintaining health. Dysregulated IL-6 is closely associated with many diseases, making it a key research and therapeutic target. Elevated IL-6 levels in the central nervous system worsen neuroinflammation in neurodegenerative diseases by activating microglia and astrocytes and releasing pro-inflammatory cytokines and neurotoxic molecules. Moreover, dysregulated IL-6 weakens the blood-brain barrier, exacerbating neuroinflammation and neuronal damage by allowing peripheral immune cells and inflammatory mediators to enter the brain. Mesenchymal stem cells (MSCs) show promise in modulating neuroinflammation by regulating IL-6 levels. They effectively suppress pro-inflammatory cytokines, including IL-6, while promoting anti-inflammatory factors. This therapeutic approach highlights the importance of targeting IL-6 and other inflammatory mediators to alleviate neuroinflammation and its adverse effects on neurological disorders. This review provides a comprehensive overview of IL-6’s involvement in neurological disorders, examining endogenous IL-6 and IL-6 derived from MSCs. We explore IL-6’s mechanisms affecting neuronal function, survival, and immune modulation in the central nervous system. Additionally, we discuss the potential of MSC-derived IL-6 in neuroregeneration and neuroprotection. By elucidating IL-6’s interplay with neurological pathologies, this review offers insights into novel therapeutic strategies targeting IL-6 signaling pathways for neurological disorders.

## 1 Introduction

Interleukin-6 (IL-6) is an indispensable pleiotropic cytokine, that plays a pivotal role in orchestrating the body’s immune response to infection, injury, or inflammation to induce and coordinate the different elements of the acute-phase response (1–3). Its multifaceted actions encompass the stimulation of activation and proliferation of diverse immune cells, including T cells, B cells, and macrophages. Additionally, IL-6 facilitates the differentiation of B cells into plasma cells, which is essential for antibody production (4–8).

Beyond its immunomodulatory functions, IL-6 also influences hematopoiesis, the intricate process of blood cell formation within the bone marrow, by promoting the differentiation of hematopoietic stem cells into various blood cell types, ensuring a balanced and functional immune system (9–12). Furthermore, IL-6 triggers the liver to produce acute- phase proteins essential for inflammation, tissue repair, and immune responses. It also contributes to fever induction during infection or inflammation by interacting with the hypothalamus, the brain region regulating body temperature (13–16).

In summary, IL-6 is a multifaceted cytokine with crucial roles in immune response modulation, inflammation regulation, and diverse physiological processes throughout the body (17). The IL-6 intricate functions underscore its significance in maintaining health and homeostasis. Dysregulation of IL-6 is closely linked to numerous diseases, emphasizing its prominence as a prime target for both research exploration and therapeutic intervention strategies (18, 19).

The clinical significance of IL-6 spans multiple domains (5). In inflammatory disorders, dysregulation of IL-6 signaling is at the core of conditions like rheumatoid arthritis (20–23), inflammatory bowel disease (24), and systemic lupus, exacerbating inflammation, that cause to tissue damage (25, 26). IL-6’s involvement in cancer extends beyond inflammation, since the IL-6 serves as “fuels” to tumor growth, promoting angiogenesis, and facilitating metastasis (27–29). By contrast, the IL-6 deficiency has been shown to exacerbate neurodegenerative disorders (30).

Given its critical role in various diseases, IL-6 and its receptors have emerged as promising therapeutic targets (31–34). However, IL-6 is naturally expressed by different mesenchymal stroma/stem cells (MSCs) populations, since its expression regulates the MSC stemness (35), *in vitro* proliferation (36) and differentiation (37, 38). Based on this, herein we summarize the IL-6 roles in pathophysiology of neurodegenerative disorders and, discussing the possible IL-6 and MSC-derived IL-6 therapeutic applications for the treatment of these diseases.

## 2 IL-6 signaling pathways

The IL-6/IL-6R axis is crucial for mediating a wide range of biological processes, including immune response modulation, inflammation regulation, and cellular proliferation (1–3). IL-6 exerts its effects through classic signaling, where it binds to the membrane-bound IL-6 receptor (mIL-6R) on target cells, and trans signaling, where it interacts with the soluble IL-6 receptor (sIL-6R) and the signal transducer gp130 on cells that do not express mIL-6R. This axis plays a pivotal role in the acute-phase response, T-cell differentiation, and B-cell maturation, contributing to both pro-inflammatory and anti-inflammatory effects (39, 40). In the central nervous system, IL-6 can activate glial cells and disrupt the blood-brain barrier, exacerbating neuroinflammation, while also promoting neuronal survival and neurogenesis. IL-6 signaling exerts influence over specific brain regions crucial for cognitive function, motor control, and emotional regulation (41, 42). Dysregulation of IL-6 signaling within these regions, such as the hippocampus, cortex, striatum, and substantia nigra, can significantly affect neurological function and contribute to the manifestation of various symptoms observed in neurological disorders (43–45). Additionally, IL-6 influences metabolic processes and is implicated in conditions like cancer, where it supports tumor growth and survival. Understanding the IL-6/IL-6R axis is essential for developing targeted therapies for a variety of diseases, including autoimmune disorders, neurodegenerative diseases, and metabolic syndromes (28, 39, 46).

**Figure 1:**
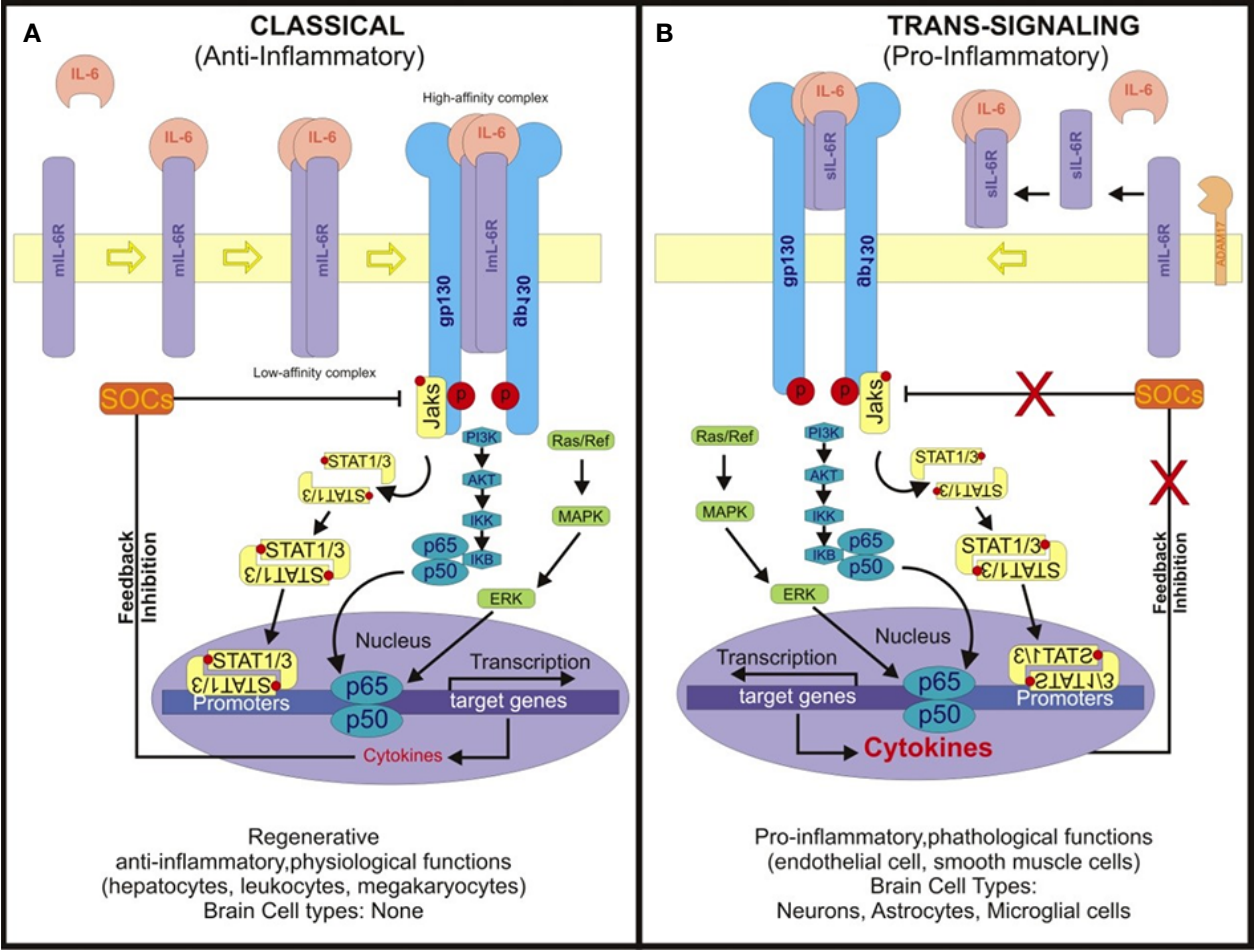
Scheme depicting the classical IL-6 and trans-signaling pathways. **(A)** In classical IL-6 signaling, IL-6 binds to membrane-boundIL-6 receptor (mIL-6R), initiating complex formation with gp130. This triggers dimerization of IL-6R, assembling a hexameric signaling complex. This complex activates intracellular cascades including JAK/STAT, MAPK/MEK-ERK, and PI3K/AKT pathways. Phosphorylated JAKs recruit and phosphorylate STAT proteins, enabling their nuclear translocation and transcriptional activation. The Classical IL-6 signaling regulates genes involved in inflammation, immune response, proliferation, and differentiation. It primarily occurs in select peripheral cell types. **(B)** The soluble IL-6 receptor (sIL-6R) assumes a pivotal role in IL-6 trans-signaling by engaging with interleukin-6 (IL-6) in the extracellular milieu. This complex formation ensues subsequent to the cleavage of the membrane-bound IL-6 receptor (mIL-6R) from cell membranes by ADAM17, thereby releasing sIL-6R. Unlike classical signaling, where IL-6 binds exclusively to mIL-6R on specific cell types, trans-signaling via the IL-6/sIL-6R complex can transpire in any cell expressing the glycoprotein 130 (gp130) receptor. Given the ubiquitous expression of gp130, this complex has the capacity to activate a plethora of signaling pathways, including the Janus kinase/signal transducer and activator of transcription (JAK/STAT), mitogen-activated protein kinase (MAPK), and phosphoinositide 3-kinase (PI3K)/Akt pathways. Such broad activation spectrum culminates in diverse cellular responses, notably amplifying JAK/STAT signaling while modulating the MAPK pathway through SOCS suppression and NF-κB, thereby fostering a pro-inflammatory milieu. This mechanism assumes paramount importance in tissues devoid of mIL-6R expression, thereby ensuring that IL-6 signaling retains its capacity to modulate gene expression and immune responses across a diverse array of cell types, including the brain, where it significantly influences inflammation and immune functions.

IL-6 signaling pathways constitute intricate networks of molecular interactions governing the biological effects of IL-6 (47–49). The initiation of IL-6 signaling commences with IL-6 binding to its specific receptor, the IL-6 receptor (IL-6R), existing in two forms: membrane-bound (mIL-6R) and soluble (sIL-6R). The classical IL-6 signaling pathway primarily operates through membrane-bound IL-6R (mIL-6R) and involves four key steps (**Figure 1**).

In addition to the classical pathway, IL-6 can initiate signaling through a trans-signaling mechanism involving soluble IL-6 receptor (sIL-6R) (48, 49). This process begins with the binding of IL-6 to its soluble receptor (sIL-6R), forming a complex. The sIL-6R is generated through proteolytic cleavage of mIL-6R or alternative splicing, releasing the extracellular domain of mIL-6R into circulation. Subsequently, the soluble IL-6/sIL-6R complex circulates freely in the bloodstream (48, 49).

In tissues lacking mIL-6R expression, the soluble IL-6/sIL-6R complex binds to the glycoprotein 130 (gp130) receptor on the cell surface. Gp130 is ubiquitously expressed on various cell types. Binding of the soluble IL-6/sIL-6R complex to Gp130 triggers receptor dimerization and activates intracellular signaling pathways (50). These signaling cascades typically involve the activation of the Janus kinase/signal transducer and activator of transcription (JAK/STAT) pathway, mitogen-activated protein kinase (MAPK) pathway, and phosphoinositide 3-kinase (PI3K)/Akt pathway (51, 52). Activation of these pathways culminates in the modulation of gene expression and cellular responses akin to the classical IL-6 signaling pathway.

To comprehensively understand the functional mechanisms depicted in **Figure 1**, it’s essential to delineate the roles of each cell type involved in the IL-6 signaling pathways. IL-6-secreting cells produce and release interleukin-6 (IL-6), a pivotal pro-inflammatory cytokine. Cells expressing membrane-bound IL-6 receptor (mIL-6R) facilitate IL-6 binding, forming a complex with glycoprotein 130 (gp130), which transduces downstream signals. ADAM17-expressing cells, such as certain immune cells, cleave mIL-6R to release soluble IL-6 receptor (sIL-6R). In turn, cells lacking mIL-6R but expressing gp130 can still respond to IL-6 via trans-signaling when the IL-6/sIL-6R complex binds to gp130. This intricate interplay among various cell types orchestrates IL-6 signaling, culminating in the activation of downstream pathways and modulation of cellular responses, including inflammation and immune reactions.

While the classic IL-6 signaling pathway is responsible for the anti-inflammatory actions, the IL-6 trans-signaling pathway contribute to pathogenic activities. Thus, they diverge notably in their cellular targets and the breadth of their biological impact across various tissues and organ systems. In the classic pathway, membrane-bound IL-6R is crucial for cell activation, while the trans-signaling pathway exploits soluble IL-6R to broaden the spectrum of target cells and biological responses.

## 3 Role of IL-6 during development and adult life

During embryonic development, interleukin-6 (IL-6) is produced by diverse cell types, including trophoblasts (53), extraembryonic endoderm (54), embryonic stem cells (ESCs) (55, 56), and mesenchymal stem cells (MSCs) (35, 36, 57), contributing to the intricate orchestration of early developmental processes. Trophoblast cells, forming the outer layer of the blastocyst during early embryonic development, are essential for implantation, placental formation, and nutrient exchange between the developing embryo and the maternal environment. Recognized as IL-6 producers, trophoblasts suggest potential regulatory functions during early embryonic development and implantation processes.

Additionally, the extraembryonic endoderm, derived from the blastocyst’s inner cell mass, significantly contributes to yolk sac formation and placental development. These cells also produce IL-6 during early embryogenesis, implicating its involvement in supporting placental growth and function (11, 58, 59).

ESCs, originating from the blastocyst’s inner cell mass, exhibit remarkable pluripotency, capable of differentiating into cell types representing all three germ layers. Under specific conditions, ESCs have been observed to produce IL-6, particularly during *in vitro* culture and differentiation procedures, suggesting a potential role for IL-6 in guiding ESC fate and development (60, 61).

Furthermore, MSCs, progenitors of various connective tissues such as bone, cartilage, and muscle, actively participate in organogenesis and tissue remodeling throughout embryonic development. IL-6 production by these cells hints at its regulatory involvement in these processes, potentially influencing tissue differentiation and morphogenesis (35–37, 57).

The diverse production of IL-6 by various cell types during embryonic development underscores its importance in regulating key developmental events and highlights its potential as a critical player in guiding embryonic growth and organogenesis.

In adult life, IL-6 plays multifaceted roles beyond its well-established functions in immune response regulation and inflammation modulation. A crucial aspect of IL-6 activity lies in its contribution to tissue repair and regeneration following injury or damage in adults. This cytokine serves as a key orchestrator in the intricate process of wound healing, facilitating the proliferation and migration of various cell types important for tissue restoration, including fibroblasts, essential for generating connective tissue; endothelial cells crucial for blood vessel formation; and immune cells that aid in tissue remodeling and defense against pathogens (62–64).

Moreover, IL-6’s involvement extends to the regeneration of specific tissues such as muscle and liver. By promoting cellular proliferation and tissue remodeling mechanisms, IL-6 aids in the restoration of structural and functional integrity in these vital organs post-injury or during pathological conditions. After skeletal muscle injury, IL-6 is dynamically released from damaged muscle fibers and promotes infiltration of immune cells within the injury site. Acting as a key regulator of muscle repair, IL-6 serves as a potent stimulator of myoblast proliferation, the precursor cells crucial for initiating muscle regeneration. Additionally, IL-6 promotes the fusion of myoblasts into multinucleated myotubes, a fundamental step in the restoration of muscle fiber integrity post-injury. Furthermore, IL-6 exerts its regenerative influence by stimulating the secretion of growth factors, including insulin-like growth factor-1 (IGF-1), which amplifies the reparative processes within the injured muscle tissue (65). Studies utilizing animal models underscore the indispensable role of IL-6 in muscle regeneration, as mice deficient in IL-6 exhibit delayed and impaired recovery, emphasizing the essential contribution of IL-6 in ordering efficient muscle repair mechanisms. Furthermore, IL-6 exerts a significant influence on neurological functions in adults (64, 66–70).

The diverse roles of IL-6 in tissue repair, neurological function, and disease pathogenesis underscore its importance as a multifunctional cytokine in adult physiology and pathology. Further understanding of its intricate mechanisms of action holds promise for the development of targeted therapeutic interventions for a range of conditions affecting human health.

## 4 IL-6 in neurological disorders

The classical IL-6 signaling pathway profoundly influences neurodegenerative diseases, playing an essential role in various aspects of their pathophysiology. Similarly, the IL-6 trans-signaling pathway holds significant relevance in these conditions. Consequently, dysregulation of IL-6 emerges as a substantial factor in various neurological disorders, including Alzheimer’s disease (AD), Parkinson’s disease (PD) (71, 72), and Huntington’s disease (HD) (30, 73, 74), Multiple Sclerosis (MS) (75, 76), Amyotrophic Lateral Sclerosis (ALS) (77, 78) an others. HD, AD, and PD are marked by the gradual deterioration of nerve cells in specific brain regions, such as the striatum in HD, the hippocampus and cortex in AD, and the substantia nigra in PD. This degeneration leads to cognitive decline and motor impairment, affecting various aspects of daily life. While they exhibit overlapping symptoms like cognitive deficits and movement issues, each has unique genetic origins and impacts individuals uniquely. Nonetheless, these conditions profoundly impact the well-being of those affected and their families, underscoring the crucial demand for efficacious treatments. These diseases were specifically chosen to exemplify IL-6’s role in the neuroinflammation process.

In Alzheimer’s disease (AD), characterized by amyloid beta (Aβ) plaque accumulation and tau pathology, IL-6 influences Aβ production and clearance. Dysregulated IL-6 signaling disrupts the delicate balance between Aβ production and clearance mechanisms, leading to toxic Aβ species accumulation and neurotoxic plaque formation (79). Another hallmark of AD is the abnormal phosphorylation and aggregation of tau protein into neurofibrillary tangles. IL-6 has also been implicated in tau pathology, affecting tau phosphorylation and aggregation processes (80). Dysregulated IL-6 signaling can contribute to tau pathology by promoting aberrant tau phosphorylation and impairing tau clearance mechanisms, which ultimately exacerbate neuronal dysfunction and degeneration (81).

Moreover, AD is characterized by chronic neuroinflammation, marked by sustained activation of microglia and astrocytes (82). IL-6 exacerbates chronic neuroinflammation, affecting the blood-brain barrier (BBB) and influencing neuronal survival. Dysregulated IL-6 signaling contributes to neuroinflammation, releasing pro-inflammatory cytokines and reactive oxygen species, further damaging neurons, and impairing cognitive function in AD (83). Additionally, IL-6 can affect the integrity of the BBB, which regulates the passage of molecules and immune cells between the bloodstream and the brain. Dysregulated IL-6 signaling compromises BBB integrity, allowing peripheral immune cells and inflammatory mediators to infiltrate the brain, further amplifying neuroinflammation and neuronal damage in AD (42, 84, 85). Furthermore, IL-6 plays a complex role in regulating neuronal survival and synaptic plasticity in the brain. While acute IL-6 signaling may promote neuroprotection and synaptic plasticity, chronic or dysregulated IL-6 signaling can lead to neuronal dysfunction and synaptic loss. This imbalance in IL-6 signaling disrupts the delicate equilibrium between neuronal survival and death, contributing to neurodegeneration in AD (86–89).

In Huntington’s disease (HD), marked by motor dysfunction, cognitive decline, and psychiatric symptoms (90), IL-6 dysregulation (30, 73) leads to several detrimental effects. First, it contributes to excitotoxicity, an overwhelming stimulation of glutamate receptors resulting in neuronal damage. IL-6 enhances glutamate release while impairing its reuptake, exacerbating excitotoxicity in HD (91, 92). Additionally, IL-6 is implicated in the dysregulation of intracellular calcium signaling, further contributing to neuronal dysfunction and cell death (93). Astrocyte dysfunction, increasingly recognized in HD pathology, is also influenced by IL-6 signaling. Dysregulated IL-6 signaling disrupts astrocyte function, impairing their ability to support neuronal health and regulate synaptic activity. This disruption in astrocyte-neuron interactions contributes to neuronal dysfunction and degeneration (94, 95). While acute IL-6 signaling may have neuroprotective and tissue repair roles, chronic or dysregulated IL-6 signaling has detrimental effects. It impairs the brain’s ability to mount effective neuroprotective and repair responses, exacerbating neuronal damage and disease progression in HD (74).

Furthermore, HD presents psychiatric symptoms like depression and anxiety, and IL-6 dysregulation has been implicated in their pathophysiology. Elevated IL-6 levels are associated with depressive symptoms in HD patients, suggesting a potential role for IL-6 in the psychiatric manifestations of the disease (73). Therefore, dysregulated IL-6 signaling plays a significant role in HD progression by fostering neuroinflammation, enhancing excitotoxicity, disrupting astrocyte function, impairing neuroprotective mechanisms, and potentially exacerbating psychiatric symptoms (96–100).

IL-6 has attracted considerable interest within the context of Parkinson’s disease (PD), a progressive neurodegenerative disorder characterized by motor manifestations such as tremors, rigidity, and bradykinesia, coupled with non-motor symptoms encompassing cognitive decline and mood disturbances (80, 101–105). Furthermore, IL-6-induced Chronic Inflammatory Response (CISR) Syndrome precipitates toxic neuronal iron accumulation, thereby contributing to synuclein-induced neurodegeneration (106).

Despite the intricate and multifaceted nature of IL-6’s precise contribution to PD, recent insights suggest its involvement in various facets of the disorder’s pathophysiology. Neuroinflammation plays a pivotal role in PD, with dysregulated IL-6 signaling contributing to the chronic inflammatory state observed in this condition. This is substantiated by elevated levels of IL-6 detected in the cerebrospinal fluid and brain tissue of PD patients (107–110).

Additionally, studies indicate that IL-6 can initiate the demise of dopaminergic neurons through inflammatory pathways and oxidative stress mechanisms, potentially contributing to the progressive loss of these neurons in PD. Furthermore, IL-6’s impact on the integrity of the blood-brain barrier (BBB) is noteworthy, facilitating the entry of peripheral immune cells and inflammatory molecules into the brain (111–115), thereby exacerbating neuroinflammation and neuronal damage in PD, as well as motor and cognitive impairment, and an increased risk of dementia in PD patients (107, 108, 116, 117).

Dysregulated IL-6 signaling significantly contributes to the pathogenesis of neurodegenerative diseases (AD, HD, and PD) by promoting neuroinflammation, exacerbating Aβ accumulation and tau pathology in AD, compromising BBB integrity, and impairing neuronal survival and synaptic plasticity. Targeted interventions aimed at IL-6 signaling pathways offer-promising strategies for managing these conditions and potentially slowing disease progression.

## 5 Mesenchymal stem cells in IL-6 regulation

Mesenchymal stem cells (MSCs) represent a versatile type of multipotent adult stem cell with the ability to differentiate into osteoblasts, chondrocytes, and adipocytes. Widely distributed in tissues like bone marrow, adipose tissue, umbilical cord blood, and dental pulp, MSCs are valued for their self-renewal capacity, making them promise in regenerative medicine and tissue engineering applications (90, 118).

While MSCs can express and secrete various cytokines, including IL-6, their IL-6 production is generally lower compared to immune cells like macrophages or T cells. However, under specific conditions, such as exposure to inflammatory stimuli or interactions with immune cells, MSCs can produce IL-6. The extent of IL-6 secretion by MSCs depends on factors like their source, culture conditions, and microenvironmental cues, emphasizing a context-dependent regulation of IL-6 production (36–38, 119–122).

MSCs have been extensively studied in clinical trials across various medical conditions, demonstrating potential from musculoskeletal disorders to autoimmune diseases and inflammatory ailments (123, 124). In the context of neuroinflammation, MSCs exhibit promise in modulating IL-6 expression. The downregulation of IL-6 by MSCs involves autocrine and paracrine signaling loops, as well as feedback regulation within the immune system (125–128).

In autocrine regulation, cells producing IL-6 respond to their own secretion, amplifying IL-6 production in response to stimuli. Paracrine signaling involves IL-6 influencing neighboring cells, leading to coordinated IL-6 expression among different cell types. Feedback mechanisms within the immune system, influenced by IL-6 signaling, further regulate IL-6 production (129–133). Additionally, IL-6 expression can be regulated by feedback mechanisms within the immune system, where IL-6 signaling influences the differentiation and activation of immune cells, thereby modulating IL-6 production (134–136).

The intricate influence of MSCs on endogenous IL-6 production is contingent upon cellular interactions and experimental parameters (36). Gu et al. (119) delineated that MSC-induced endogenous IL-6 release resulted in upregulating of IL-6R and p-STAT3 levels in astrocytes subjected to oxygen and glucose deprivation. Notably, a conspicuous elevation in the Bcl-2 to Bax ratio, pivotal downstream factors of the STAT3 signaling pathway, was observed. This investigation elucidated the neuroprotective impact of MSCs transplantation in neonatal hypoxic-ischemic brain damage rats, partly mediated by IL-6, enhancing the anti-apoptotic profile of injured astrocytes via the IL-6/STAT3 signaling pathway. While MSCs have demonstrated their capability to suppress IL-6 production by immune cells such as macrophages and T cells through paracrine signaling and immunomodulatory mechanisms (137, 138), MSC-derived IL-6 has also been shown to stimulate or modulate the activity of other immune cells, thereby influencing endogenous IL-6 levels (139, 140).

MSCs exhibit anti-inflammatory effects, affecting IL-6 levels across various contexts and can downregulate IL-6 production by suppressing the activation of immune cells. This was evident in a study involving inflammatory bowel disease (IBD) in mice, where MSC administration led to diminished levels of pro-inflammatory cytokines, including IL-6, in inflamed colon tissue (141, 142). Mechanistically, MSCs manifest their anti-inflammatory effects through the secretion of factors such as transforming growth factor-beta (TGF-β) and prostaglandin E2 (PGE2), as well as by fostering the generation of regulatory T cells (Tregs) (143, 144).

## 6 MSCs and IL-6 in neuroinflammation and neuroprotection

In the intricate landscape of neuroinflammation, both MSCs and IL-6 wield substantial influence, jointly shaping the pathophysiology of diverse neurological disorders.

**Figure 2:**
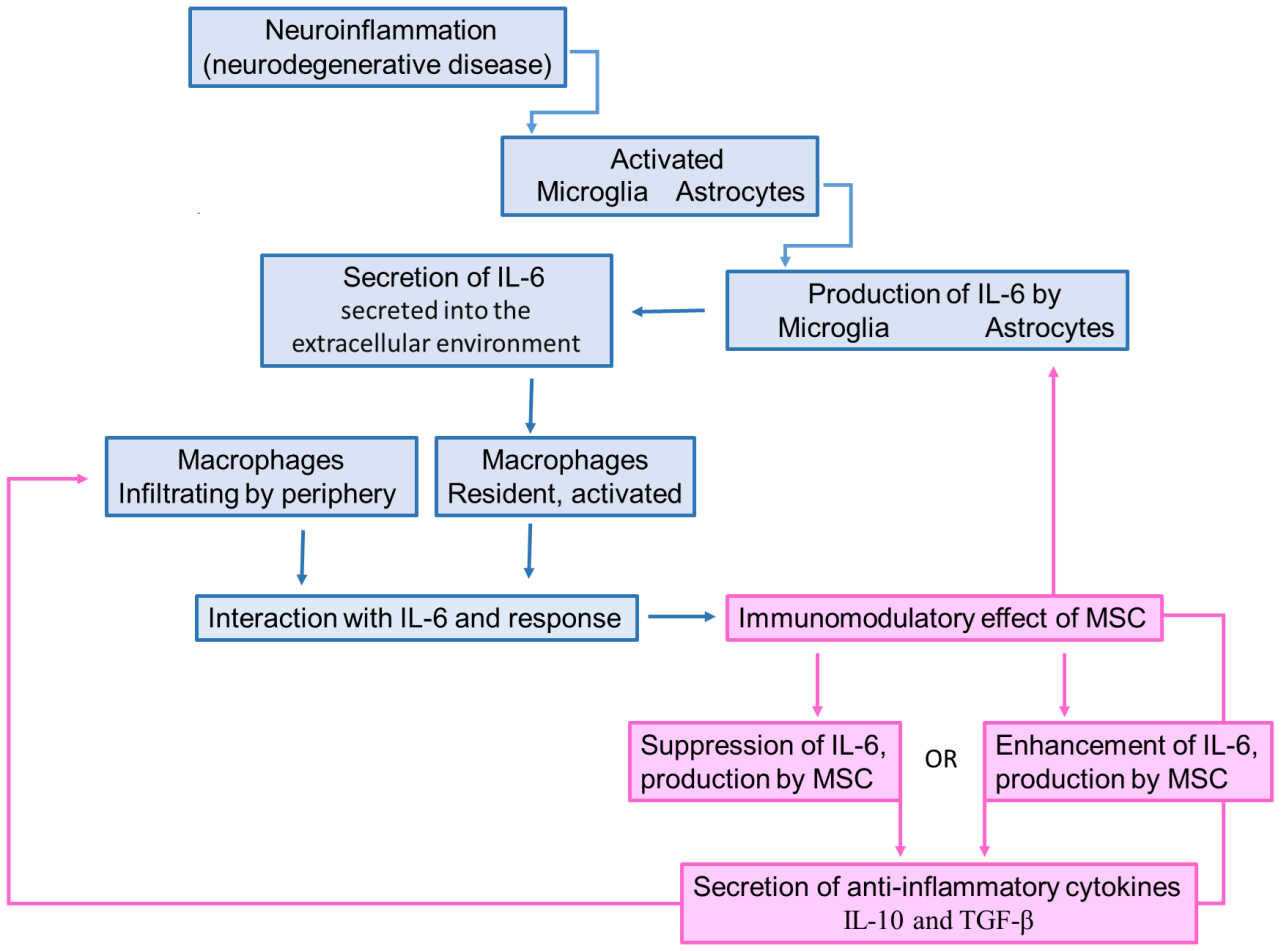
Schematic illustration describing the IL-6 role in neurodegeneration and how the MSC-secreted IL-6 can promote immunomodulation. Neuroinflammatory process caused by the bioaccumulation of misfolded proteins promotes the activation of microglia and astrocytes. Once activated, these cells produce and secrete IL-6, which attracts monocytes to be differentiated into macrophages. However, the MSC transplantation increases the IL-6 levels, leading to the production of IL-10 and TGF-β by macrophages, which suppress the microglia/astrocyte activation.

When introduced into neuroinflammatory conditions, MSCs exhibit the capacity to downregulate the expression of IL-6 by activated microglia, astrocytes, and infiltrating immune cells within the central nervous system (CNS), as illustrated in **Figure 2**. This regulatory effect is achieved through the secretion of anti-inflammatory factors such as IL-10 and TGF-β, which effectively inhibit the synthesis and release of IL-6 by immune cells. Additionally, MSCs foster the generation of Tregs, contributing to an additional layer of suppression on IL-6 production and the attenuation of neuroinflammation (145–147).

In the complex realm of immunomodulation, MSCs play a crucial role in balancing pro-inflammatory and anti-inflammatory forces by regulating cytokine production, notably IL-6 (**Figure 2**). Both the suppression and enhancement of IL-6 production by MSCs influence the secretion of anti-inflammatory cytokines. When MSCs suppress IL-6 production, they create a favorable environment for anti-inflammatory cytokine secretion by dampening the pro-inflammatory response. This displays MSCs’ multifaceted immunomodulatory capabilities, where reduced IL-6 levels pave the way for anti-inflammatory mediators. Conversely, when MSCs enhance IL-6 production, they introduce complexity to immune regulation. Despite IL-6’s pro-inflammatory reputation, it can exhibit anti-inflammatory effects in certain contexts. By increasing IL-6 levels, MSCs may trigger a regulatory response, leading to the secretion of anti-inflammatory cytokines to counteract inflammation. In both scenarios, achieving the desired outcome relies on a delicate balance influenced by cellular signals and contextual cues. MSCs act as pivotal orchestrators in this intricate dance of immunomodulation, guiding the secretion of anti-inflammatory cytokines amidst the dynamic landscape of inflammation and immune response.

Recent research underscores that endogenous MSCs transplantation significantly enhances cognitive function and sustains brain health in neonatal rats with hypoxic-ischemic brain damage (HIBD), owing to the immune-regulating abilities of MSCs (119). Notably, this study reveals a substantial boost in IL-6 release compared to other cytokines upon MSC transplantation. When MSCs with reduced IL-6 expression were used, positive effects on behavior and brain activity were notably diminished correlating with decreased IL-6 levels in the hippocampus. This study sheds light on MSCs enhancing the survival of injured astrocytes through the IL-6/STAT3 signaling pathway, indicating IL-6 as a key mediator in the neuroprotective effects, emphasizing the critical role of IL-6 in the neuroprotective effects of MSCs transplantation in neonatal HIBD rats partly rely on IL-6, promoting the survival of injured astrocytes via the IL-6/STAT3 signaling pathway (119).

Conversely, IL-6 secreted by exogenous cells can influence the immunomodulatory properties of MSCs and influence their behavior within the CNS. Studies suggest that IL-6 enhances the immunosuppressive function of exogenous MSCs and facilitates their migration to inflammatory sites in the CNS (148, 149). However, prolonged exposure to high levels of IL-6 may jeopardize the therapeutic potential of MSCs and compromise their regenerative capacity. IL-6 signaling has the potential to disrupt the equilibrium between pro-inflammatory and anti-inflammatory factors secreted by exogenous MSCs, leading to dysregulated immune responses and persistent neuroinflammation (150).

The modulation of IL-6 signaling pathways, coupled with leveraging the immunomodulatory prowess of MSCs, emerges as a compelling avenue for developing therapeutic strategies aimed at alleviating neuroinflammation and fostering neurological recovery in neurodegenerative diseases and neurological injuries. Current research endeavors, as highlighted by Gu et al. (119), Wang et al. (151), and Kitzberger et al. (122) are diligently exploring the efficacy of MSC-based therapies and IL-6 inhibitors in the realm of neuroinflammatory disorders. These studies seek not only to unravel the therapeutic potential of such interventions but also to refine treatment strategies for patients grappling with these complex conditions.

The therapeutic promise extends to MSC-derived IL-6, operating synergistically with other bioactive factors secreted by MSCs. This collaborative action holds substantial potential for amplifying the effectiveness of MSC-based treatments in neurological disorders. Through mechanisms involving neuroprotection, neuroregeneration, and immunomodulation within the CNS, IL-6 plays a crucial role in enhancing the overall efficacy of MSC therapies. Strategic targeting of IL-6 signaling pathways, as already demonstrated (152–155), introduces innovative dimensions in neuroprotection and neurorepair.

In specific contexts, IL-6 derived from MSCs showcases neuroprotective properties by fortifying neuronal survival and providing a shield against various insults, including oxidative stress, excitotoxicity, and inflammatory cytokines (156, 157). Through intricate intracellular signaling cascades, particularly via the Janus kinase (JAK)-signal transducer and activator of transcription (STAT) pathway, IL-6 actively reinforces neuronal resilience and viability (158, 159).

Beyond these fundamental roles, IL-6 assumes a crucial position in modulating synaptic plasticity, a process essential for learning and memory consolidation. Its regulatory influence on the expression and functionality of neurotransmitter receptors, synaptic proteins, and signaling molecules (160, 161). Additionally, IL-6 actively promotes neurite outgrowth and axonal regeneration, facilitating crucial neuronal connectivity and repair within the injured or diseased CNS (162–164). Furthermore, IL-6 modulates neurotransmitter release and neuronal excitability, underscoring its multifaceted role in neurological function and pathology (165–167).

Thus, IL-6, and MSC-derived IL-6 emerges as a key player in both the pathophysiology of neurological disorders and potential therapeutic interventions. Its involvement in immunomodulation, tissue repair, and neuroprotection emphasizes its therapeutic potential in conditions such as stroke, traumatic brain injury, and neurodegenerative diseases.

## 7 Conclusions

The collaborative interaction between MSCs and IL-6 in neuroinflammation is crucial for understanding the pathophysiology of neurological disorders and developing therapeutic strategies to mitigate neuroinflammation and promote neurological recovery in various neurodegenerative diseases and neurological injuries.

The diverse roles of IL-6 in immunomodulation, tissue repair, neuroprotection, and synaptic plasticity highlight its promising application in neurological conditions ranging from stroke to traumatic brain injury and neurodegenerative diseases.

Current studies on the efficacy of MSC-based therapies and IL-6 inhibitors aim to elucidate their therapeutic potential and optimize treatment strategies for patients with these conditions.

IL-6 derived from MSCs, along with other bioactive factors secreted by them, promises to enhance the therapeutic efficacy of MSC-based treatments in neurological disorders, contributing to neuroprotection, neuroregeneration, and immunomodulation in the CNS. In this regards, strategic targeting of IL-6 signaling pathways can further amplify the effectiveness of MSC-based interventions, paving the way for innovative approaches in neuroprotection and neurorepair.

## Author contributions

AR: Writing – review & editing, Validation, Formal analysis. ÁS: Writing – review & editing, Visualization, Validation, Formal analysis. IK: Writing – review & editing, Writing – original draft, Visualization, Validation, Supervision, Project administration, Methodology, Investigation, Funding acquisition, Formal analysis, Conceptualization.
